# Using an analogical reasoning framework to infer language patterns for negative life events

**DOI:** 10.1186/s12911-019-0895-8

**Published:** 2019-08-28

**Authors:** Jheng-Long Wu, Xiang Xiao, Liang-Chih Yu, Shao-Zhen Ye, K. Robert Lai

**Affiliations:** 1 School of Big Data Management, Soochow University, Taipei City, Taiwan; 2 Department of Computer Science and Engineering, Yuan Ze University, Taoyuan City, Taiwan; 3 Innovation Center for Big Data and Digital Convergence, Yuan Ze University, Taoyuan City, Taiwan; 4 College of Mathematics and Computer Science, FuZhou University, FuZhou City, China; 5 Department of Information Management, Yuan Ze University, Taoyuan City, Taiwan

**Keywords:** Negative life event, Language pattern mining, Analogical reasoning

## Abstract

**Background:**

Feelings of depression can be caused by negative life events (NLE) such as the death of a family member, a quarrel with one’s spouse, job loss, or strong criticism from an authority figure. The automatic and accurate identification of negative life event language patterns (NLE-LP) can help identify individuals potentially in need of psychiatric services. An NLE-LP combines a person (subject) and a reasonable negative life event (action), e.g. <parent:divorce> or < boyfriend:break_up>.

**Methods:**

This paper proposes an analogical reasoning framework which combines a word representation approach and a pattern inference method to mine/extract NLE-LPs from psychiatric consultation documents. Word representation approaches such as skip-gram (SG) and continuous bag-of-words (CBOW) are used to generate word embeddings. Pattern inference methods such as cosine similarity (COSINE) and cosine multiplication similarity (COSMUL) are used to infer patterns.

**Results:**

Experimental results show our proposed analogical reasoning framework outperforms the traditional methods such as positive pairwise mutual information (PPMI) and hyperspace analog to language (HAL), and can effectively mine highly precise NLE-LPs based on word embeddings. CBOW with COSINE of analogical reasoning is the best word representation and inference engine. In addition, both word embeddings and the inference engine provided by the analogical reasoning framework can further be used to improve the HAL model.

**Conclusions:**

Our proposed framework is a very simple matching function based on these word representation approaches and is applied to significantly improve HAL model mining performance.

## Background

The prevalence of clinical depression has increased rapidly in recent years, and the onset of depression can be triggered or exacerbated by negative or stressful life events, such as the death of a family member, a quarrel with one’s spouse, job loss, or conflict with an authority figure. Such negative life events (NLE) are associated with the onset of depressive episodes, such as anxiety [1], suicide attempts [2] and bulimic symptoms [3]. Many online services have been developed to assess mental health and provide treatment. Users interact with these websites by writing about their feelings and recent negative life experiences, and these utterances are referred to as negative life event language patterns (NLE-LPs). These text-based messages are then manually reviewed by professional psychologists who provide diagnosis and recommendations, but this is a labor-intensive process and responses may take several days, and this lengthy delay may have dire consequences, especially for those with suicidal tendencies. Therefore, many researches are used the online text-based messages to mining its affective meaning [4, 5].

Automating the identification of NLE-LPs in social media texts could reduce the delay in diagnosis and intervention response. However, NLE-LPs are unstructured expressions made up of non-continuous word combinations. For example, there are two NLE-LPs such as <*brother*:*lovelorn* > and < *brother*:*resigned* > in a consultation sentence as “*Brother* has *broken up*, and *resigned* his job in government”. For the two NLE-LPs in this case, “brother” is the subject, and “lovelorn” and “resigned” are two actions which combine to constitute two negative life events. NLE-LPs are also combinations of nouns and verbs. Analogical reasoning is a significant logic component of human intelligence [6–8]. It is a reasoning method which infers similarities between things which share certain attributes, giving the two items similar status or properties. For example, *α* and *β* are a subject and action, and the *α*^∗^ is another subject. The analogy suggests that the relationship between *α* and *β* is similar to the relationship between *α*^∗^ and *β*^∗^, thus *α*, *β* and *α*^∗^ can be used to refer to *β*^∗^, which is an inference target. Analogical reasoning has recently attracted increased attention in the field of artificial intelligence because an analogy is a very basic form of logical inference [9]. It has been widely applied to natural language processing (NLP) tasks such as question answering [10–12], word segmentation [13], latent relational analysis [14, 15], and recommendation systems [16].

The relationship between two words is measured according to relational or attributional similarity. Relational similarity calculates the degree of similarity between two patterns (or pairs), called an analogy [17]. Attributional similarity calculates the degree of similarity between two words, called synonyms. In information theory, mutual information (MI) and pointwise mutual information (PMI) are used to measure the similarity between two things (or patterns) and simulate human memory [18–20]. In general, they calculate similarity based on co-occurrence frequency, and this simple approach is widely applied to NLP tasks [21, 22]. Cosine similarity (COSINE) is also used to measure the degree of similarity between two vectors [23]. It has been applied to hesitant fuzzy linguistic term sets for financial performance evaluation [24]. Pre-trained word embeddings have been used for analogy tasks, and it can improve identification performances in semantic and syntactic problems [25, 26]. The word embeddings are word representations trained using raw text data and can be applied in many classification tasks [27–30]. Word embeddings have also been applied to infer word relations in analogy tasks using multiplication and division, instead of addition and subtraction [31]. The analogy method has been used to improve English to Chinese language translation [32]. In addition, over the past decade the traditional association rule mining algorithm has been used to mine language patterns using the co-occurring relationship of words [33, 34]. Association rule mining (ARM) has also been used to generate seed language patterns from a small labeled NLE corpus, using the distributed semantic model to discover expanded language patterns from a large unlabeled corpus for sentence classification [35, 36]. An advanced method, evolutionary reasoning algorithms with hyperspace analog to language (HAL) is used to iteratively induce additional relevant patterns from a seed pattern set [37]. Existing methods such as ARM and HAL are used to address many issues but do not consider the larger syntax-based semantic information. Therefore, we propose a framework which obtains word representations to improve pattern inference performance in NLE-LP mining tasks.

This paper proposes an analogical reasoning framework to mine NLE-LPs using word representation and pattern inference. There are two word representation approaches including skip-gram (SG) and continuous bag-of-words (CBOW) [25, 26]. Two methods of pattern inference (cosine similarity and COSINE multiplication similarity (COSMUL) [31, 38]) are used to create vector space models for word analogies problems. This paper has three contributions: (1) using the distributed word embeddings to capture semantic relationships from a large corpus to facilitate NLE-LP mining. (2) The analogical reasoning pattern inference is first used to extract NLE-LPs based on word embedding. (3) The analogical reasoning framework based on word embeddings can improve NLE-LP mining performance.

For the first contribution, word embedding is a new distributed word representation learning method based on neural network architectures. Compared to traditional methods that represent a word using a high-dimensional sparse vector, word embedding focuses on learning low-dimensional dense vectors for words by leveraging contextual information from corpora. Such representations have been proven to efficiently capture both semantic and syntactic information from very large datasets [25, 26].

For the second contribution, we extend the analogical reasoning framework to address language pattern mining tasks by considering the pattern structure <*subject*:*action*>. That is, given a < *subject*:*action* > pattern and another subject, the analogical reasoning framework can infer a proper action to be combined with the subject to constitute a new pattern.

For the third contribution, the use of the word embeddings and inference engine provided by the analogical reasoning framework outperforms traditional language pattern mining methods such as the positive pairwise mutual information (PPMI) and HAL model. In addition, the analogical reasoning framework can further be used to improve the HAL model by replacing the HAL word representation with word embeddings and the HAL inference scheme with analogical reasoning.

The remainder of this paper is organized as follows. Existing methods describes the overall system framework. Seed NLE-LP annotation introduces our proposed negative life event language pattern mining method which combines word representation approaches with pattern inference methods. PPMI model explains the generation of the NLE-LP dataset and summarizes and discusses the experimental results. Conclusions and directions for future work are presented in HAL model.

## Existing methods

**Fig. 1:**
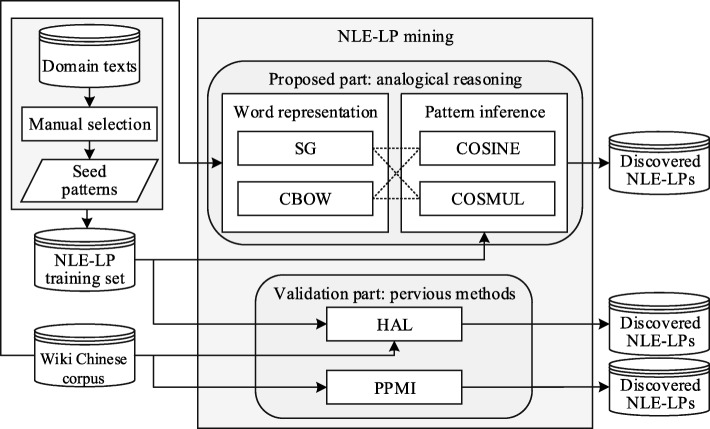
NLE-LP mining process

The overall system framework is shown in Fig. 1. First, a seed NLE-LP annotation processing uses manual annotation to identify NLE-LPs as queries from a domain text corpus. Second, NLE-LP mining is divided into a proposed part and a validation part. For the validation part, we use the previous HAL model and the positive pointwise mutual information model to extract NLE-LPs. The experimental results section compares mining performance for the NLE-LP task among the different methods.

To mine NLE-LPs, this section first introduces seed NLE-LP annotation, previous mining methods such as PPMI and HAL, and our proposed analogical reasoning framework. Given the seed NLE-LP <*α* : *β*>, the object of analogical reasoning is to infer another mined NLE-LP as <*α*^∗^ : *β*^∗^> where *α* and *α*^∗^ are known subjects, *β* is a known action, and *β*^∗^ is the action we want to infer using the proposed framework. Therefore, the query is defined as <*α* : *β* >  :  :  < *α*^∗^ :  ? >.

### Seed NLE-LP annotation

To extract the seed NLE-LPs, each seed NLE-LP was divided into a subject set and an action set. We designed a logical relationship matrix *LR* which is action by subject. The values of the *LR* matrix were provided by three domain expert annotators based on the following principle: if there exists a logical relationship between a subject and an action, then make a symbol at the intersection of subject and action, otherwise, no logical relationship exists. The annotation process by the three experts produced three *LR* matrices. To combine the three *LR* matrices by element-wise counting, if the symbol count is equal to 3, the corresponding subject and action are a seed NLE-LP. Table 1 shows the annotation example of a *LR* matrix. For example, both “*be_sick*” and “*quarrel*” have a logical relationship for all subjects. In addition, the four actions “divorce”, “*break_up*”, “*drop_out*” and “*resign*” have a logical relationship with some subjects.

**Table 1:** Example of seed NLE-LPs annotation

		Subject				
		*parents*	*boyfriend*	*classmate*	*colleague*	*friend*
Action	*be_sick*	✓	✓	✓	✓	✓
	*quarrel*	✓	✓	✓	✓	✓
	*divorce*	✓				
	*break_up*		✓			
	*drop_out*		✓	✓		✓
	*resign*	✓	✓		✓	✓

For the NLE-LP mining problem, an analogical inference query assumes the form of <*α* : *β* >  :  :  < *α*^∗^ : *β*^∗^> where *β*^∗^ is the target word (action) to be inferred. In addition, we designed two experimental problems in which, if the two subjects of *α* and *α*^∗^ are the same category (e.g., *older_brother* and *younger_brother*) this problem is called a within-category; otherwise it is called an across-category. Table 2 shows NLE-LP examples of queries for within-category and across-category problems.

**Table 2:** NLE-LP example of query and answer

Problem	<*α* : *β* > : : < *α*^∗^ : *β*^∗^>	<*α*^∗^ : *β*^∗^>
Within category	<*older_brother*:*drop_out*>::<*younger*:?>	<*younger*:*quit_school*>
	<*classmate*:*sneer*>::<*teacher*:?>	<*teacher*:*satirize*>
Across category	<*friend*:*betray*>::<*teacher*:?>	<*teacher*:*abandon*>
	<*wife*:*bicker*>::<*colleague*:?>	<*colleague*:*quarrel*>

To evaluate the language patterns discovered by different word representations, a standard answer must be established. In this experiment, a standard answer is defined such that the action *β*^∗^ is a negative life event which could reasonably occur in conjunction with the subject *α*^∗^.

### PPMI model

In the fields of data mining or information retrieval, pointwise mutual information is usually used to measure the correlation between the two concepts (word). PMI as expressed by Eq. (1) to compute the information between *x* and *y*.

1 $$ PMI\left(x,y\right)=\log \frac{p\left(x,y\right)}{p(x)p(y)} $$

where *p*(*x*) and *p*(*y*) respectively denote the probability of objects *x* and *y* appearing in the corpus. *p*(*x*, *y*) denotes the probability of *x* and *y* simultaneously appearing in a specific context. According to probability theory, if *x* and *y* are independent, then the value of *PMI*(*x*, *y*) is equal to 0. Otherwise, the value of *PMI*(*x*, *y*) is positive or negative. To avoid an insufficiently large rate of co-occurrence, we apply common positive pointwise mutual information to measure the relevance of two words, the PPMI is defined by Eq. (2).

2 $$ PPMI\left(x,y\right)=\left\{\begin{array}{l}0,\kern4em \mathrm{if}\  PMI\left(x,y\right)\le 0\\ {} PMI\left(x,y\right),\kern0.5em \mathrm{if}\  PMI\left(x,y\right)>0\end{array}\right. $$

where the *PPMI*(*x*, *y*) values are directly calculated from the preprocessed wiki Chinese corpus, and then applied to measure their mutual correlation.

### HAL model

**Fig. 2:**
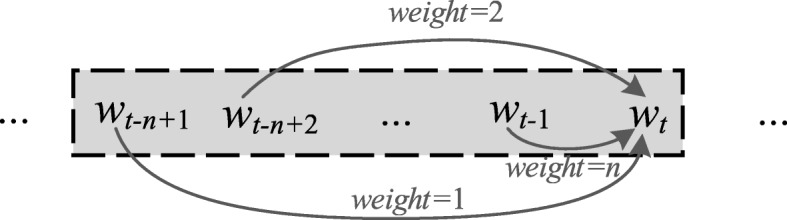
Weighting scheme of the HAL model

HAL uses a high-dimensional semantic space [39, 40] and the word representation of HAL is called the HAL-VEC. This model uses a vector to represent each word in the vocabulary. Each dimension denotes the weight of a word in the context of the target word. The weights are calculated by considering the syntactic properties, such as the location and distance of words. In principle, a word that is contextually closer to the target word will have a greater weight, while those further away will have a low weight. To capture information for co-occurring words, an observation window length must be set to establish an effective scope. For example, for a target word *w*_*t*_, the window size is *n*. There are *n*-1 preceding words need to compute its weights such as from *w*_*t-n* + 1_ to *w*_*t-*1_ Therefore, the weight of the first word *w*_*t-n* + 1_ in this window length is set to 1 and the previous word is set to *n*. Following this principle, we can slide the window over the entire sentence to calculate the word vector for each word. Figure 2 shows the weighting scheme of the HAL model.

HAL also is an information inference used to discover information flows using high dimensional conceptual space such HAL-VEC [41, 42], and is called the HAL-INF. HAL-INF uses the characteristics of HAL semantic space to capture the semantic features from the context of the word using a co-occurrence matrix constructed from the whole corpus. HAL-INF can then be used to infer semantically similar words by vector comparison. It is also a kind of analogical reasoning. Therefore, we can use the HAL-INF method to mine NLE-LPs. The degree of similarity *sim*(*β*^∗^, *λ*) of the HAL model is determined by Eq. (3).

3 $$ sim\left({\beta}^{\ast },\lambda \right)=\frac{\sum \limits_{i\in \left( QP\left(\lambda \right)\cap QP\left(V\left({\beta}^{\ast}\right)\right)\right)}V{\left({\beta}^{\ast}\right)}_i}{\sum \limits_{j\in QP\left(\lambda \right)}{\lambda}_j} $$

where *λ* is a vector and denotes the calculation result of three given vectors by *λ* = *V*(*β*) − *V*(*α*) + *V*(*α*^∗^). This definition ensures that a majority of the most important quality properties on word vectors are captured. Here, we use a threshold *δ* to filter the position of a dimension which satisfies the *δ*. The numerator calculates the accumulation of weights of those quality properties appearing in both *λ* and *β*^∗^. The denominator is the sum of all quality property weights of *v* such as *λ* and *β*^∗^. The *QP* is an index set and calculated by Eq. (4) and Eq. (5).

4 $$ QP(v)=\underset{k}{\arg }{v}_k>\delta $$

5 $$ \delta =\frac{1}{\left\Vert v\right\Vert}\sum \limits_k{v}_k $$

where *v* is word vectors and *δ* is scale.

The NLE-LPs mining performances of the PPMI and HAL baseline methods will compare to our proposed analogical framework in the experimental results section.

## Method

### Overview

Figure 1 shows our proposed analogical reasoning framework in the proposed part and infers NLE-LPs in a pipeline process including word representation and pattern inference. Two word presentation approaches are trained from the Wiki Chinese corpus and domain text corpus: CBOW and SG. Two pattern inference methods are then used to infer corresponding NLE-LPs: COSINE and COSMUL. The analogical reasoning processes are presented in Algorithm 1; through the algorithm we can obtain the final NLE-LP set. The detailed computations of word representation approach and pattern inference method are described in the following sections.

#### Word representation approach

The two word representation approaches include skip-gram and continuous bag-of-words.

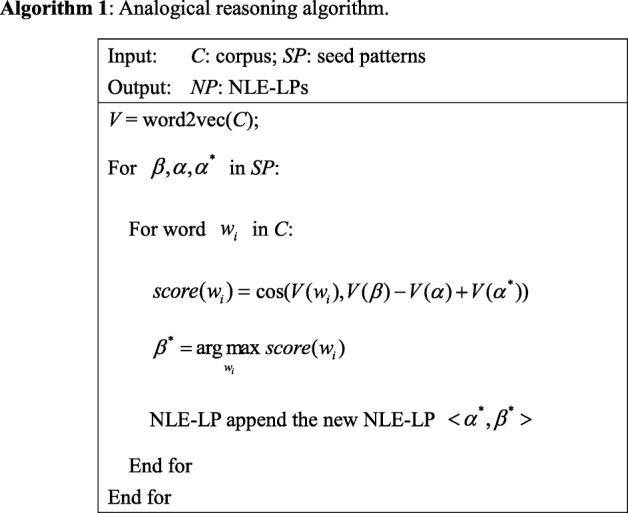



##### Skip-gram (SG) and continuous bag-of-words (CBOW)

The neural network language model (NNLM) was formally proposed by Bengio et al. [43] to construct a language model through a three-layer neural network architecture. Mikolov et al. [25, 26] proposed two new architectures for learning distributed representations of words: skip-gram and continuous bag-of-words. The two models are simplified neural network language models, removing the hidden layer, and changing from a neural network architecture to the log-linear architecture, greatly reducing computational complexity and increasing training efficiency on large datasets. The CBOW model is similar to the feedforward NNLM, where the hidden layer is removed and the projection layer is shared for all words. All words in context have the same weights for the impact on the probability of current word occurrence, and the projection is not influenced by the lack of consideration of word order. The CBOW model predicts the current word based on the text while Skip-gram uses each word as an input and predicts words within a certain range before and after the current word. SG considers that non-continuous words have a weaker relationship with the current word than those close to it, so distant words are given less weight. Since the two models can effectively train from a large corpus and obtain a distributed word representation, we use them to learn word embeddings from the Chinese Wikipedia corpus.

For the CBOW model, given the context *C* = {*w*_*t* − (*n* − 1)/2_, .., *w*_*t* − 1_, *w*_*t* + 1_, …, *w*_*t* + (*n* − 1)/2_} of the current word *w*_*t*_, the hidden feature **h** is calculated using the mean for each word in context *C* as input, defined by Eq. (6).

6 $$ \mathbf{h}=\frac{1}{\left\Vert C\right\Vert}\sum \limits_{j=1}^{\left\Vert C\right\Vert }V\left({w}_j\right) $$

where the *w*_*j*_ denotes the word vector of the *j*th word in context *C*. *V* ∈ *ℜ*^*d*^ denotes the weight of the word vector as trainable parameters. We then compute the output vector from the input as **h**, defined by Eq. (7):

7 $$ \mathbf{u}=\mathbf{wh} $$

where the **w** ∈ *R*^*v* × *d*^ denotes the trainable weight of the output layer.

For the SG model, given the context *C* of the target word *w*_*t*_, the target word is moved to the input layer and the context word is moved to the output layer. Therefore, in the SG model, the context words are the target words.

#### Pattern inference method

Based on word representation, there are two methods to infer NLE-LPs. The query includes four words: *α*, *β*, *α*^∗^ and *β*^∗^. We use the analogical relationship of *α*, *β* and *α*^∗^ to infer *β*^∗^. The <*α*^∗^ : *β*^∗^> infers the NLE-LP. The word in the vocabulary is represented as a vector using either word representation approach for analogical reasoning by vector calculation. The relationship expressed for analogical reasoning is mathematically defined by Eq. (8):

8 $$ V\left(\beta \right)-V\left(\alpha \right)\approx V\left({\beta}^{\ast}\right)-V\left({\alpha}^{\ast}\right) $$

where *V*(⋅) denotes the word vector of (⋅), the target word vector *V*(*β*^∗^) can be obtained by Eq. (9):

9 $$ V\left({\beta}^{\ast}\right)=V\left(\beta \right)-V\left(\alpha \right)+V\left({\alpha}^{\ast}\right) $$

The problem of analogical reasoning can be transformed into a problem of calculating the similarity between a candidate and given words. The analogy methods can also use different methods for calculating similarity. For the analogy query <*α* : *β* >  :  :  < *α*^∗^ : *β*^∗^>, the aim is to find a word *β*^∗^ based on a subject *α*^∗^ which is most similar to <*α* : *β*>. A general form of similarity *sim* is defined by Eq. (10):

10 $$ similarity= sim\left({\beta}^{\ast },\left\{\alpha, \beta, {\alpha}^{\ast}\right\}\right) $$

There are two methods for calculating vector similarity, and a detailed process for calculating the similarity of the two methods is as follows:

##### Cosine similarity (COSINE)

First, we adopt the most common function as cosine similarity. The similarity is 1 when the angle between the two vectors is equal to 0, and 0 when the angle is 90 degrees. The cosine similarity is defined by Eq. (11).

11 $$ sim\left({\beta}^{\ast },\left\{\alpha, \beta, {\alpha}^{\ast}\right\}\right)=\cos \left(V\left({\beta}^{\ast}\right),V\left(\beta \right)-V\left(\alpha \right)+V\left({\alpha}^{\ast}\right)\right) $$

##### Cosine multiplication similarity (COSMUL)

According to Levy et al. [31], the Eq. (8) is equivalent to Eq. (12).

12 $$ sim\left({\beta}^{\ast },\left\{\alpha, \beta, {\alpha}^{\ast}\right\}\right)=\cos \left(V\left({\beta}^{\ast}\right),V\left(\beta \right)\right)-\cos \left(V\left({\beta}^{\ast}\right),V\left(\alpha \right)\right)+\cos \left(V\left({\beta}^{\ast}\right)V\left({\alpha}^{\ast}\right)\right) $$

Therefore, Eq. 7 is revised from an additive to a multiplicative combination:

13 $$ sim\left({\beta}^{\ast },\left\{\alpha, \beta, {\alpha}^{\ast}\right\}\right)=\frac{\cos \left(V\left({\beta}^{\ast}\right),V\left(\beta \right)\right)\times \cos \left(V\left({\beta}^{\ast}\right)V\left({\alpha}^{\ast}\right)\right)}{\cos \left(V\left({\beta}^{\ast}\right),V\left(\alpha \right)\right)+\varepsilon } $$

where *ε* is a very small value in order to prevent division by zero, and setting to 0.001.

## Results

This section introduces the experimental NLE-LP dataset, implementation mining models, evaluation metrics, experimental results and discussion.

### NLE-LP dataset

Many online resources exist for the discussion of psychiatric issues and depression. Psychiatric consultation records contain meaningful descriptions about stress, anxiety, and other negative emotions. No personal information on the users was provided. The content of these texts include many negative life events language patterns, but these are typically difficult to detect automatically due to the use of natural language expressions. NLE-LPs can appear in both continuous and discontinuous text strings, and multiple NLE-LPs may be included or overlap in a sentence. To identify potential NLE-LPs, we first analyze the raw sentence’s semantics. Table 3 shows two real world NLE-LPs by matching the sentences in the corpus with the mined language patterns.

**Table 3:** Examples of original sentences with NLE-LPs

No.	Original sentence	NLE-LP
1	It makes me very uncomfortable when my *parents* only see their own view point when they *criticize* me.	<*parents*:*criticize*>
2	My *boyfriend* of 3 years suddenly *broke up* with me and I’m really sad.	<*boyfriend*:*break_up*>

NLE-LPs are considered only to consist of noun-verb pairs. The noun is the person, and the verb is related to negative events taken from the daily life corpus. NLE-LPs must have a logical relationship with the person and negative life events; that is, the pairing of the subject and the action must be logically reasonable. To facilitate annotators in identifying logically reasonable pairs of subjects and actions, we create a logical relationship matrix LR (as shown in Table 1) in the annotation process. For example, the annotation results presented in Table 1 show that some actions (e.g., divorce) are not well paired with some subjects (e.g., boyfriend, classmate, etc.). The initial set of 132 seed NLE-LPs is obtained by manual review of 500 sentences from a psychiatric text for appropriate subject/action pairs (respectively a total of 54 subjects and 152 negative life events). As shown in Table 4, all seed NLE-LPs are divided into five categories including family, love, school, work and social. Seed NLE-LP identify 6002 reasonable NLE-LPs out of 8208 (152*54) LPs.

**Table 4:** Distribution of seed NLE-LPs

NLE category	Count	Ratio
Work	9	6.8%
Love	20	15.2%
School	21	15.9%
Social	23	17.4%
Family	59	44.7%

To capture more possible NLE-LP queries, we apply a knowledge-based as an ontology by extended-HowNet ontology^1^ (E-HowNet). As shown in Table 5, the action words are expanded, e.g., the term “complain” can be expanded to “beef” and “blame”.

**Table 5:** Example of expanded word as action using E-HowNet

Seed action	Expanded action
Complain	Beef, blame
Deceive	Lie, cheat
Denounce	Condemn, denounce

The knowledge-based expansion operation produces a total of 318,106 queries. Each of the five categories (family, love, school, work and social) are subdivided into 5 subject pair subsets. The query set is divided into 25 subsets according to the category to which the two subjects belong. There are 5 within-category analogy query sets of the same class, and the other 20 are across-category analogy query sets. For the analogical reasoning experiments, we randomly select 1000 NLE-LPs from each subset and another 2000 NLE-LPs as a test set without duplication from each subset. Finally, there are a total of 25,000 NLE-LPs in the training set and 50,000 NLE-LPs in the test set.

### Implementation details

We implement PPMI and HAL with the four proposed analogical reasoning models: SG with COSINE, CBOW with COSINE, SG with COSMUL, CBOW with COSMUL. We also implement the four improved HAL models: HAL-VEC with COSINE, HAL-VEC with COSMUL, SG with HAL-INF and COBW with HAL-INF.

#### Baseline methods

- **PPMI**: This is a traditional language pattern mining model used as a baseline using previous PPMI method
- **HAL**: This is another baseline language pattern mining model using previous HAL-based model

#### Analogical reasoning models

Here, we present four NLE-LP mining models using COBW and SG word representation approaches and COSINE and COSMUL pattern inference methods.

- **CBOW + COSINE**: This model combines CBOW word representation and COSINE pattern inference.
- **SG + COSINE**: This model combines SG word representation and COSINE pattern inference.
- **CBOW + COSMUL**: This model combines CBOW word representation and COSMUL pattern inference.
- **SG + COSMUL**: This model combines SG word representation and COSMUL pattern inference.

### Improved-HAL models

We use an analogical approach of COSINE and COSMUL, along with word embedding of SG and COBW to improve the HAL model.

- **HAL-VEC + COSINE**: This model combines the HAL-VEC word vector and COSINE pattern inference, which the COSINE replacing the HAL-INF.
- **HAL-VEC + COSMUL**: This model combines the HAL-VEC word vector and COSMUL for pattern inference, which the COSMUL replacing the HAL-INF.
- **CBOW + HAL-INF**: This model combines the CBOW word representation and HAL-INF pattern inference, which the CBOW replacing the HAL-VEC.
- **SG + HAL-INF**: This model combines SG word representation and HAL-INF pattern inference, which the SG replacing the HAL-VEC.

### Evaluation metrics

To evaluate NLR-LP mining performance, we propose two metrics to evaluate all experiments of the NLE-LP mining problem, including mean reciprocal rank (*MRR*) and precision (*prec*@*n*).

#### MRR

*MRR* is a general measure of quality and is the average of the reciprocal ranks of results for a sample of queries *Q* [44]. The *MRR* is defined by Eq. (14).

14 $$ MRR=\frac{1}{\left|Q\right|}\sum \limits_{q=1}^Q\frac{1}{{\operatorname{rank}}_q} $$

where *Q* denotes the query set, and rank_*q*_ denotes the rank position of the first relevant NLE-LP for the *q-*th query.

#### Prec@n

*prec*@*n* is a top *n* measuring for a query. We select *n* items as NLE-LPs from the intersection of two sets which are extracted NLE-LPs and relevant NLE-LPs (gold standard NLE-LPs). The *prec*@*n* is defined by Eq. (15).

15 $$ prec@n=\frac{\left\Vert NS\left(\left\{\mathrm{extracted}\ \mathrm{NLE}\hbox{-} \mathrm{LPs}\right\}\right)\cap \left\{\mathrm{relevant}\ \mathrm{NLE}\hbox{-} \mathrm{LPs}\right\};n\Big)\right\Vert }{n} $$

where ‖⋅‖ denotes the number of documents in the set, and *NS* denotes a selection operation for selecting *n* NLE-LPs, given intersection set and *n*.

### Comparative results

To obtain experimental results, the candidate words are filtered by part-of-speech, and only nouns and verbs are respectively considered as relevant for the person and negative life events. The target of NLE-LP < subject:action> is <noun:verb>. Table 6 presents the results of different methods for NLE-LP mining on within-category and across-category problems. Two baseline methods (PPMI and HAL) are used along with four analogical reasoning methods: COBW + COSINE, SG + COSINE, CBOW + COSMUL and SG + COSMUL. Prior to NLE-LP mining, the NLE-LP analogy query set is divided into two parts: a training set used to adjust the parameters for the word representation training process, and a test set used to mine NLE-LPs. Each method is evaluated using 50,000 examples in the test set, where 10,000 examples are used for the within-category setting and 40,000 for the across-category setting.

**Table 6:** Comparative results of different methods for NLE-LP mining task

Method		Within-category			Across-category		
		*prec*@5	*prec*@10	*MRR*	*prec*@5	*prec*@10	*MRR*
Traditional induction	PPMI	–	–	–	0.1296	0.1241	0.2589
	HAL	0.2034	0.1842	0.3921	0.2116	0.1872	0.3820
Analogical Reasoning	CBOW + COSINE	0.4904	0.4300	0.7287	0.4684	0.4129	0.7037
	SG + COSINE	0.4345	0.3824	0.6683	0.4215	0.3705	0.6683
	CBOW + COSMUL	0.4272	0.3809	0.6654	0.4004	0.3589	0.6278
	SG + COSMUL	0.3961	0.3516	0.6286	0.3751	0.3321	0.6197

We run experiments with two baselines of NLE-LP mining methods. The PPMI model as a baseline method is used for NLE-LP mining using only term frequency and term co-occurrence but without the word representation approach. Therefore, we directly compute the relationship between subject word and action word by PPMI. For a given subject, the composition of the NLE-LP is determined by calculating the value of the PPMI with other action words. PPMI is the process of deducing one word from another. Compared to the analogical reasoning query set, there is only one subject for each query in pattern statistical induction, and the definitions of the standard answers are consistent with the answers to the analogical reasoning query. The mining performance of the PPMI model is not well suited for NLE-LP extraction, providing only 0.1296 *prec*@5, 0.1241 *prec*@10 and 0.2589 *MRR*. The HAL model is another baseline method used to mine NLE-LPs using the HAL-VEC and HAL-INF. This model obtained 0.2034 *prec*@5, 0.1842 *prec*@10 and 0.3921 *MMR* on the within-category problem, and 0.2116 *prec*@5 0.1872 *prec*@10 and 0.3820 *MMR* on the across-category problem. Therefore, the HAL model presents only a very small difference for the three metrics in the two problems. However, the HAL model outperformed the PPMI model on the across-category problem.

Among the four mining methods in our proposed analogical reasoning framework, COBOW + COSINE provides the best mining performance with results of 0.4904 *prec*@5, 0.43 *prec*@10 and 0.7287 *MRR* on the category analogy problem, and 0.4684 *prec*@5, 0.4129 *prec*@10 and 0.7037 *MRR* on the non-category analogy problem. SG + COSINE provides the second best mining performance with results of 0.4345 *prec*@5, 0.3824 *prec*@10 and 0.0.6683 *MRR* on the category analogy problem, and 0.4215 *prec*@5, 0.3.705 *prec*@10 and 0.6683 *MRR* on the non-category analogy problem. CBOW + COSMUL follows with 0.4272 *prec*@5, 0.3809 *prec*@10 and 0.6654 *MRR* on the category analogy problem, and 0.4004 *prec*@5, 0.3589 *prec*@10 and 0.6278 *MRR* on the non-category analogy problem. SG + COSMUL provides the lowest mining performance.

Based on the performance of these six methods, four methods of the analogical framework outperformed the traditional PPMI and HAL models, because the two word embeddings set by COBW and SG were trained using data from the Chinese version of Wikipedia which provides semantic and syntactic relationship information. Therefore, COSINE and COSMUL based on word embeddings can obtain sufficient information to infer pattern similarity. In addition, two pattern inference methods are considered to compute the similarity by all values of two word vectors instead of partial values of two word vectors such as in the HAL model.

### Improved-HAL results

This section presents the four implemented improved-HAL methods to evaluate whether our proposed analogical reasoning framework improves the HAL model. The four improved-HAL methods are used to mine NLE-LPs on 25 query subsets, with results shown in Table 7. First, we use COSINE and COSMUL to replace the HAL-INF inference approach of the HAL model. The COSINE pattern inference method of the analogical reasoning framework produces results of 0.4279 *prec*@5, 0.3738 *prec*@10 and 0.6495 *MRR* for the within-category problem, and 0.4232 *prec*@5, 0.3709 *prec*@10 and 0.6598 *MRR* on the across-category problem. The COSMUL pattern inference method slightly underperforms COSINE. Table 6 shows the COSMUL pattern inference method also does not outperform COSINE. In the HAL-VEC word vector of HAL model, the CBOW and SG word representation approaches are used to replace HAL-VEC. The CBOW of analogical reasoning framework produces results of 0.3482 *prec*@5, 0.3112 *prec*@10 and 0.575 *MRR* on the within-category problem, and 0.3311 *prec*@5, 0.2999 *prec*@10 and 0.5525 *MRR* on the across-category problem. The SG word representation approach slightly underperforms CBOW. In terms of improving word representation and pattern method for the HAL model, the two pattern inference methods provide better improvement than the two word representation approaches. This is because COSINE and COSMUL are applied to compute similarity using all values of the two vectors. By replacing HAL-INF, which only uses partial vector values, with either COSINE or COSMUL we can compute the full range of information. However, all improved-HAL methods outperform traditional the HAL and PPMI models for the two NLE-LP mining problems.

**Table 7:** Comparative results of improved-HAL methods for NLE-LP mining task

Method	Within-category			Across-category		
	*prec*@5	*prec*@10	*MRR*	*prec*@5	*prec*@10	*MRR*
HAL	0.2034	0.1842	0.3921	0.2116	0.1872	0.3820
Improved-HAL						
HAL-VEC + COSINE	0.4279	0.3738	0.6495	0.4232	0.3709	0.6598
HAL-VEC + COSMUL	0.4270	0.3737	0.6494	0.4203	0.3702	0.6530
CBOW + HAL-INF	0.3482	0.3112	0.5750	0.3311	0.2999	0.5525
SG + HAL-INF	0.3395	0.2990	0.5715	0.3377	0.2965	0.5776

### Sensitivity analysis for vector dimension and windows size

**Fig. 3:**
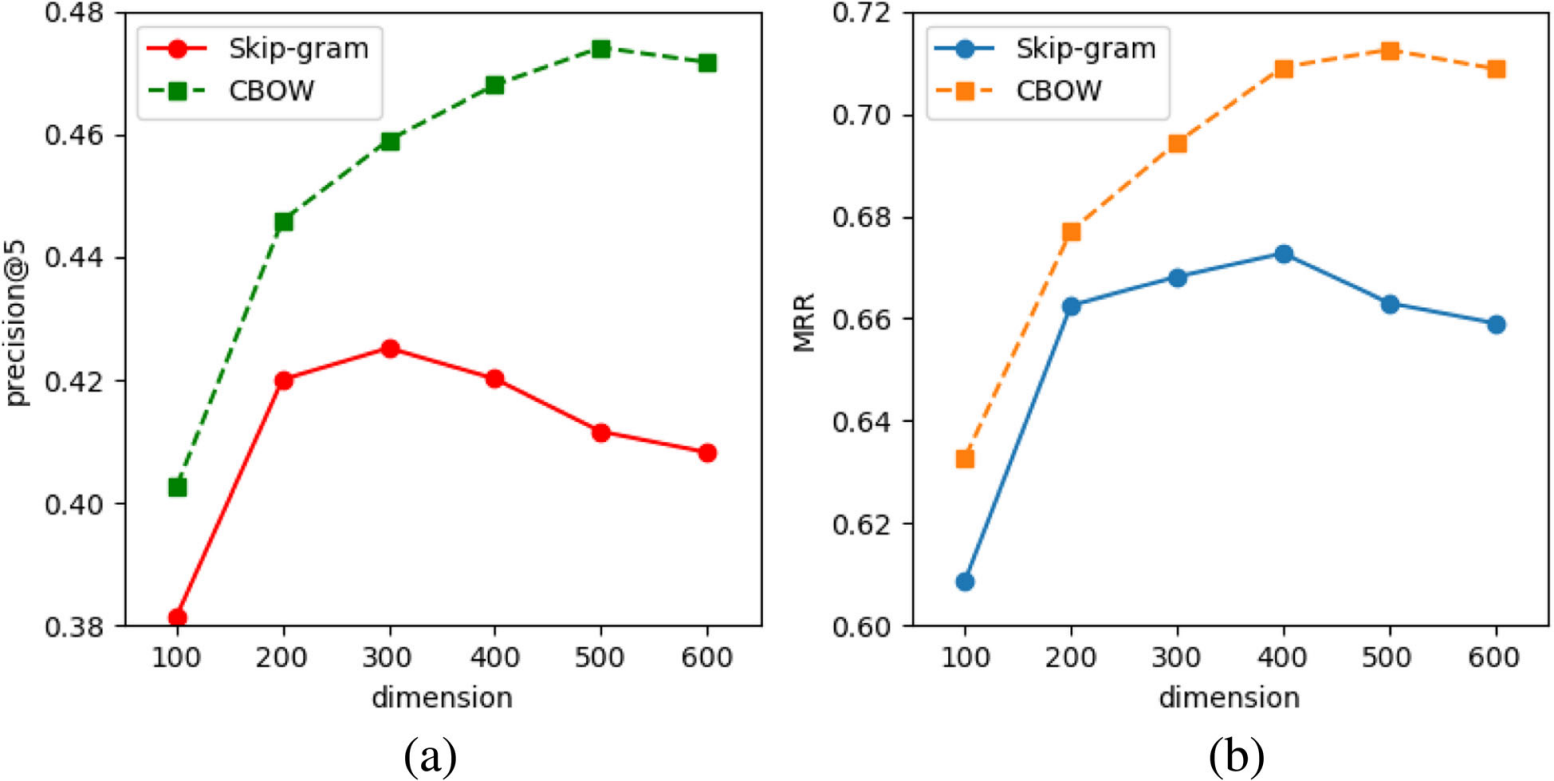
Performance compassion using different dimension sizes for SG and CBOW with COSINE pattern inference on the across-category analogy problem. Performances: (**a**) precision, (**b**) MMR

The dimension size of the hyper-parameter is a major issue in the SG and CBOW models, and is used in the training set to determine the optimal vector dimension size. Figure 3 shows the results of dimension size sensitivity analysis for the SG and CBOW models. The horizontal axis represents the dimension size and the vertical axis represents the mining performance of the two metrics. Six dimension sizes are used from 100 to 600 for SG and CBOW. In Fig. 3(a), the curve of precision@5 shows that the best dimension is obtained using 500 and 300 on the COBW model and 300 on the SG model. The *MRR* curve in Fig. 3(b) shows that optimal mining performance is achieved using 500 dimensions in the SG model and 400 dimensions in the CBOW model. In addition, the COBW model consistently outperforms the SG model according to *precsion*@5 and *MRR*.

**Fig. 4:**
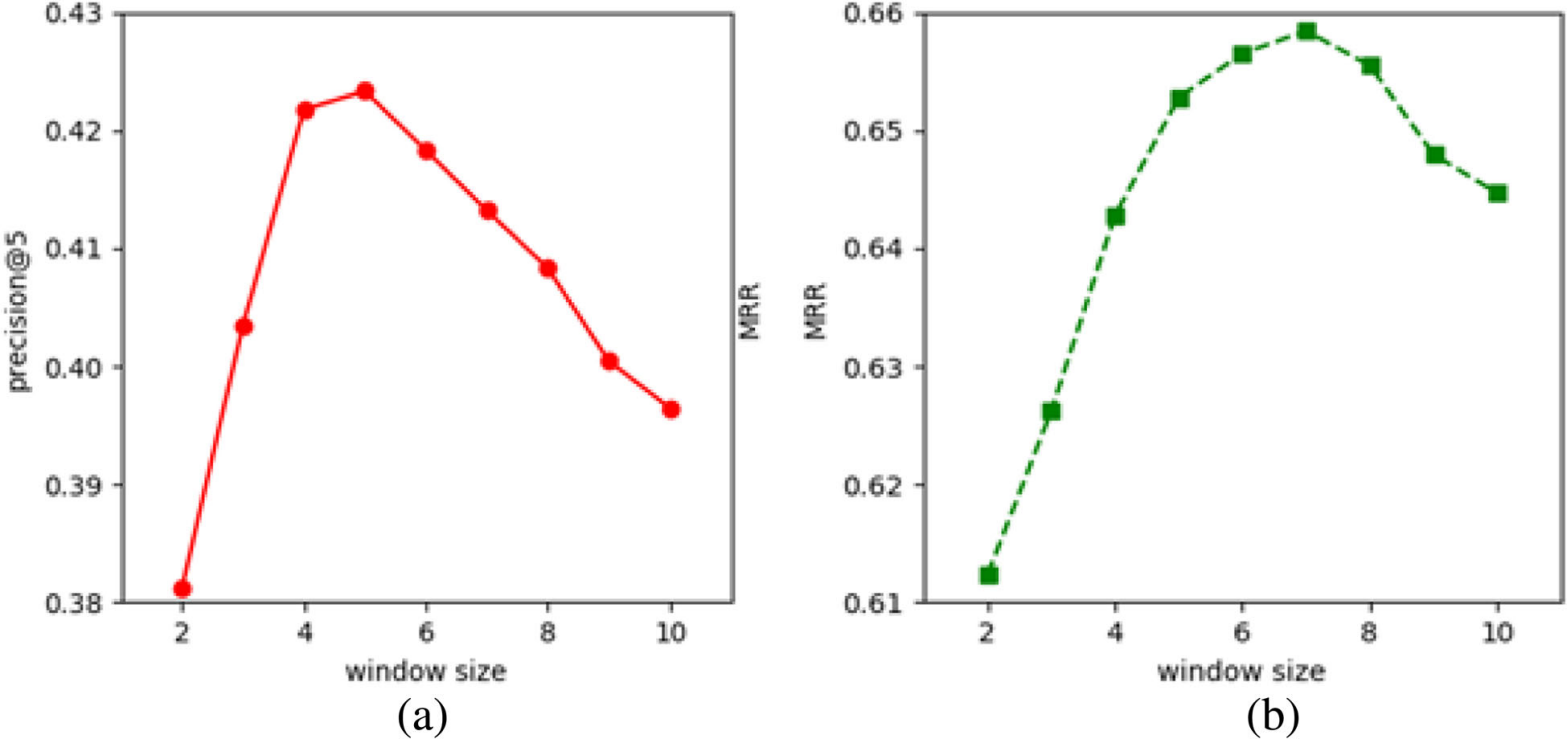
Performance compassion using different window sizes for the HAL-VEC word representation approach with the COSINE pattern inference method on the across-category analogy problem. Performances: (**a**) precision, (**b**) MMR

The window size is another important hyper-parameter when building word vectors using the HAL-VEC word representation approach. We apply different windows sizes ranging from two to ten to evaluate the mining performance using the COSINE pattern inference method on the non-category analogy problem. Figure 4 shows the two performance curves for nine different window sizes using the HAL-INF pattern inference model. In Fig. 4(a), the highest score for the *precision*@5 curve is 0.4232 with a window size of five. For the curve of *MRR* scores in Fig. 4(b), the highest *MRR* score is 0.6598 with a window size of 7.

## Discussion

Experimental results demonstrate the proposed approach provides acceptable performance for the NLE-LP mining problem. The four combined analogical reasoning models outperform the PPMI and HAL models for NLE-LP mining task. Optimal NLE-LP mining performance is obtained using the CBOW word representation approach with the COSINE pattern inference method. In this paper, the analogical reasoning framework is used to effectively mine NLE-LPs. SG and CBOW are popular low-dimensional distributed word embedding approaches, HAL-VEC is a high-dimensional semantic space, but replacing HAL-VEC with either SG or CBOW outperforms the traditional HAL model. Analogical reasoning is the most common cosine similarity calculation for two semantic and syntactic tasks. COSMUL is a modified cosine similarity calculation method, which does not match cosine’s mining performance. The HAL-INF of HAL is an inspired information inference model, but replacing HAL-INF with either COSINE or COSMUL outperforms traditional the HAL model. Among these two pattern inference methods, the common cosine similarity COSINE provides the best performance for the NLE-LP mining task.

## Conclusion

This paper proposes a novel analogical reasoning framework to mine negative life event language patterns using word representation with pattern inferences. This is the first instance of using the analogical reasoning framework to infer NLE-LP. The framework of analogical reasoning uses two word representation approaches and two pattern inference methods which outperform the traditional PPMI model and HAL model. PMMI is very simple and quickly mines NLE-LPs, but with poor inference performance. While word representation training requires extra time to learn word embeddings, it accounts for semantic relationships in the corpus and thus is more effective in mining NLE-LPs. The COSINE method outperforms other pattern inference methods such as COSMUL and HAL-INF. It also provides a very simple matching function based on these word representation approaches. In addition, COBW, SG, COSINE and COSMUL are applied to significantly improve the HAL model mining performance.

Mining performance for the NLE-LP task is still low and, to obtain high-precision semantic relationships, we propose using the advanced word representation approach such Doc2Vec, which consider document information to learn word embeddings. In addition, pattern inference precision can be further improved by using more professional depression corpora which include additional depressive words for training word embedding. However, there are two constraints of our proposed analogical reasoning framework in NLE-LP task, one is word representation learning needs a larger corpus related to NLE because NLE-LPs are logically reasonable pair, such as an incorrect sample < boyfriend:divorce>. Second, the pattern inference needs significant word vectors for inference because all word vectors should be embedded in highly precise semantic relationships using the word representation approach.

## Data Availability

The datasets generated during the current study are available in the https://github.com/jlwustudio/nle-lp_arf.

## Abbreviations

CBOW: Continuous bag-of-words
COSINE: Cosine similarity
COSMUL: Cosine multiplication similarity
HAL: Hyperspace analog to language
MI: Mutual information
MRR: Mean reciprocal rank
NLE: Negative life events
NLE-LP: Negative life event language patterns
NLP: Natural language processing
PMI: Pointwise mutual information
PPMI: Positive pointwise mutual information
prec@n: precision
SG: Skip-gram

## Footnotes

1 E-HowNet: an ontology for Chinese knowledge representation. http://ehownet.iis.sinica.edu.tw/

## References

[1] Drake KE, Sheffield D, Shingler D (2011). The relationship between adult romantic attachment anxiety, negative life events, and compliance. Personal Individ Differ.

[2] Bakhiyiabc CL, Jaussentc I, Beziatc S, Cohende R, Gentyacd C, Kahnde JP, Leboyerdf M, Vaoug PL, Guillaumeacd S, Courtetacd P (2017). Positive and negative life events and reasons for living modulate suicidal ideation in a sample of patients with history of suicide attempts. J Psychiatr Res.

[3] Bodella LP, Smitha AR, Holm-Denomab JM, Gordonc KH, Joinera TE (2011). The impact of perceived social support and negative life events on bulimic symptoms. Eat Behav.

[4] Wang J, Yu LC, Lai KR, Zhang X (2016). Dimensional sentiment analysis using a regional CNN-LSTM model. 54th Annual Meeting of the Association for Computational Linguistics (ACL 2016).

[5] Yu LC, Lee LH, Hao S, Hu J, Lai KR (2016). Building Chinese affective resources in Valence-Arousal dimensions. 15th Annual Conference of the North American Chapter of the Association for Computational Linguistics: Human Language Technologies (NAACL-HLT 2016).

[6] Gentner D, Holyoak KJ, Kokinov BN (2001). The Analogical Mind: Perspectives from Cognitive Science.

[7] Cambria E, Gastaldo P, Bisio F, Zunino R (2015). An ELM-based model for affective analogical reasoning. Neurocomputing.

[8] Melis E, Veloso M (1998). Analogy in problem solving. Handbook of practical reasoning: computational and theoretical aspects.

[9] Prade H, Richard G (2014). A short introduction to computational trends in analogical reasoning. Computational Approaches to Analogical Reasoning: Curr Trends.

[10] Toba HA, Manurung M, HM. (2012). Predicting answer location using shallow semantic analogical reasoning in a factoid question answering system. 26th Pacific Asia Conference on Language, Information, and Computation (PACLIC-12).

[11] Tu X, Feng D, Wang XJ, Zhang L (2012). Analogical reasoning for answer ranking in social question answering. IEEE Intell Syst.

[12] Chaudhri VK, Heymans S, Overholtzer A, Wessel M (2014). Large-scale analogical reasoning. 29th Conference on Artificial Intelligence (AAAI-14).

[13] Hug N, Prade H, Richard G (2015). Experimenting analogical reasoning in recommendation. 1st International Symposium on Methodologies for Intelligent Systems (ISMIS-14).

[14] Duc NTB, Ishizuka D, M. (2010). Using relational similarity between word pairs for latent relational search on the web. 2010 IEEE/WIC/ACM International Conference on Web Intelligence and Intelligent Agent Technology (WI-IAT-10).

[15] Liang CLZ (2012). Chinese analogy search considering multi relations. 3rd International Conference on Cloud and Service Computing (CSC-12).

[16] Zheng ZW, Lepage Y, Y. (2015). Chinese word segmentation based on analogy and majority voting. 29th Pacific Asia Conference on Language, Information and Computation (PACLIC 2015).

[17] Turney PD (2006). Similarity of semantic relations. Comput Linguist.

[18] Tang B, Cao H, Wu Y, Jiang M, Xu H (2013). Recognizing clinical entities in hospital discharge summaries using structural support vector machines with word representation features. BMC Med Inform Decis Mak.

[19] Rahmaninia M, Moradi P (2018). OSFSMI: online stream feature selection method based on mutual information. Appl Soft Comput.

[20] Recchia G, Jones MN (2009). More data trumps smarter algorithms comparing pointwise mutual information with latent semantic analysis. Behav Res Methods.

[21] Terra E, Clarke CL (2003). Frequency estimates for statistical word similarity measures. 2003 Conference of the North American Chapter of the Association for Computational Linguistics on Human Language Technology (NAACL-03).

[22] Van de Cruys T (2011). Two multivariate generalizations of pointwise mutual information. 2011 Workshop on Distributional Semantics and Compositionality (DiSCo-11).

[23] Pramanik S, Biswas P, Giri BC (2017). Hybrid vector similarity measures and their applications to multi-attribute decision making under neutrosophic environment. Neural Comput & Applic.

[24] Dong JY, Chen Y, Wan SP (2018). A cosine similarity based QUALIFLEX approach with hesitant fuzzy linguistic term sets for financial performance evaluation. Appl Soft Comput.

[25] Mikolov T, Chen K, Corrado G, Dean J (2013). Efficient estimation of word representations in vector space. International Conference on Learning Representations (ICLR-13).

[26] Mikolov T, Sutskever I, Chen K, Corrado G, Dean J (2013). Distributed representations of words and phrases and their compositionality. 26th Advances in neural information processing systems (NIPS-13).

[27] Du J, Zhang Y, Luo J, Jia Y, Wei Q, Tao C, Xu H (2018). Extracting psychiatric stressors for suicide from social media using deep learning. BMC Med Inform Decis Mak.

[28] Choia H, Chob K, Bengioc Y (2017). Context-dependent word representation for neural machine translation. Comput Speech Lang.

[29] Turner CA, Jacobs AD, Marques CK, Oates JC, Kamen DL, Anderson PE, Obeid JS (2017). Word2Vec inversion and traditional text classifiers for phenotyping lupus. BMC Med Inform Decis Mak.

[30] Yu LC, Wang J, Lai KR, Zhang X (2018). Refining word embeddings using intensity scores for sentiment analysis. IEEE/ACM trans. Audio Speech Lang Process.

[31] Levy O, Goldberg Y, Ramat-Gan I (2014). Linguistic regularities in sparse and explicit word representations. 18th Conference on Computational Natural Language Learning (CoNLL-14).

[32] Qiu L, Zhang Y, Lu Y (2015). Syntactic dependencies and distributed word representations for Chinese analogy detection and mining. 2015 Conference on Empirical Methods on Natural Language Processing (EMNLP-15).

[33] Chien JT (2006). Association pattern language modeling. IEEE Trans Audio Speech Lang Process.

[34] Mendes AC, Antunes C (2009). Pattern mining with natural language processing: An exploratory approach. 6th International Conference on Machine Learning and Data Mining in Pattern Recognition.

[35] Yu LC, Chan CL, Wu CH, Lin CC (2009). Mining association language patterns for negative life event classification. Joint Conference of the 47th Annual Meeting of the Association for Computational Linguistics and the 4th International Joint Conference on Natural Language (ACL-IJCNLP-09).

[36] Yu LC, Chan CL, Lin CC, Lin IC (2011). Mining association language patterns using a distributional semantic model for negative life event classification. J Biomed Inform.

[37] Yu LC, Wu CH, Yeh JF, Jang FL (2008). HAL-based evolutionary inference for pattern induction from psychiatry web resources. IEEE Trans Evol Comput.

[38] Linzen T (2016). Issues in evaluating semantic spaces using word analogies. The 1st Workshop on Evaluating Vector-Space Representations for NLP.

[39] Lund K, Burgess C (1996). Producing high-dimensional semantic spaces from lexical co-occurrence. Behav Res Methods Instrum Comput.

[40] Burgess C, Livesay K, Lund K (1998). Explorations in context space words, sentences, discourse. Discourse Process.

[41] Song D, Bruza PD (2001). Discovering information flow suing high dimensional conceptual space. 24th Annual International ACM SIGIR Conference on Research and Development in Information Retrieval (SIGIR-01).

[42] Song D, Bruza PD (2003). Towards context sensitive information inference. J Assoc Inf Sci Technol.

[43] Bengio Y, Ducharme R, Vincent P, Jauvin C (2003). A neural probabilistic language model. J Mach Learn Res.

[44] Radev DR, Qi H, Wu H, Fan W (2002). Evaluating web-based question answering systems. Third International Conference on Language Resources and Evaluation (LREC-02).

